# Evaluating the Impact of Advanced Trauma Life Support (ATLS) Training in Reducing Preventable and Potentially Preventable Deaths: A Mixed-Methods Cohort Study

**DOI:** 10.7759/cureus.81681

**Published:** 2025-04-03

**Authors:** Sumeet Kumar, Siddharth Singh, Ritu Singh, Gagan Gunjan, Anand Dev

**Affiliations:** 1 Surgery, Indira Gandhi Institute of Medical Sciences, Patna, IND; 2 Emergency Medicine, Indira Gandhi Institute of Medical Sciences, Patna, IND; 3 Critical Care Medicine, Indira Gandhi Institute of Medical Sciences, Patna, IND; 4 Internal Medicine, Rajendra Institute of Medical Sciences, Ranchi, IND

**Keywords:** advanced trauma life support (atls), india, preventable deaths, survival analysis, trauma mortality, trauma training

## Abstract

Background

Trauma remains a leading global cause of mortality, particularly in low-resource settings. Advanced Trauma Life Support (ATLS) training offers a standardized approach to trauma care, yet its impact in India remains underexplored. This study evaluates the effectiveness of ATLS training in reducing preventable trauma-related deaths and improving clinical decision-making at a tertiary care center in Bihar, India.

Methods

This mixed-methods cohort study was conducted from October 2021 to December 2024 and included 200 trauma patients aged ≥18 years. Patients were divided into two groups: pre-ATLS (retrospective; October 2021 to July 2024) and post-ATLS (prospective; August 2024 to December 2024). Mortality rates, trauma management errors, and survival outcomes were analyzed using Kaplan-Meier survival curves, chi-square tests, logistic regression models, and Cox proportional hazards regression. Additionally, structured interviews with healthcare providers supplemented the quantitative findings. Statistical analyses were performed using IBM SPSS Statistics for Windows, Version 20.0 (Released 2019; IBM Corp., Armonk, NY, USA) and R software, Version 4.1.2 (R Foundation for Statistical Computing, Vienna, Austria), with significance set at p < 0.05.

Results

ATLS training significantly reduced preventable deaths (from 30% to 15%) and potentially preventable deaths (from 40% to 25%). Trauma management errors, including delayed diagnoses and protocol deviations, also decreased post-training. Kaplan-Meier analysis demonstrated improved survival, while Cox regression identified ATLS training as an independent predictor of survival (HR = 0.62, 95% CI: 0.45-0.85, p = 0.003). Healthcare providers reported greater confidence in trauma assessment but highlighted concerns regarding accessibility, cost, and the need for refresher courses.

Conclusions

ATLS training enhances trauma care by reducing preventable mortality, minimizing errors, and boosting provider confidence. Expanding ATLS programs, particularly in resource-limited settings, is recommended. Future research should focus on long-term patient outcomes and the cost-effectiveness of ATLS implementation.

## Introduction

Trauma remains one of the leading causes of morbidity and mortality globally. According to WHO, injuries are responsible for approximately 5.8 million deaths each year, accounting for nearly 10% of global mortality [1]. Road traffic accidents alone result in more than 1.35 million deaths annually and are the leading cause of death among individuals aged 5 to 29 years [2]. The burden of trauma is especially severe in low- and middle-income countries, where emergency care systems are often underdeveloped [3].

India carries a disproportionate share of the global trauma burden, accounting for nearly 10% of all road traffic fatalities worldwide. Every year, more than 150,000 deaths and 500,000 serious injuries occur in India due to road accidents alone [4]. Bihar, one of the most populous states in India, reflects this public health crisis, with over 10,000 road accidents reported in 2021, leading to more than 3,000 deaths [5]. In addition to road traffic accidents, other significant contributors to trauma include falls, burns, and interpersonal violence, all of which add to the load of preventable mortality [6].

The Advanced Trauma Life Support (ATLS) program, developed by the American College of Surgeons, offers a structured and standardized approach to trauma care, emphasizing rapid assessment and resuscitation [7]. Evidence shows that healthcare professionals trained in ATLS demonstrate improved confidence and efficiency in trauma management, contributing to reduced rates of preventable and potentially preventable deaths [8]. The program has gained global adoption, with multiple studies supporting its effectiveness in improving survival rates and reducing treatment delays [9].

Despite its global recognition, the impact of ATLS in resource-limited settings such as India, particularly in states like Bihar, has not been extensively studied. Understanding its effectiveness in such environments is essential for strengthening trauma care systems, improving patient outcomes, and minimizing preventable deaths [10].

This study was designed to address that gap by evaluating the impact of ATLS training on trauma-related mortality at a tertiary care center in Bihar. Specifically, the study aimed to determine whether an increase in ATLS-trained personnel translated into improved trauma care outcomes and fewer management errors. The objectives included examining the relationship between ATLS training and the incidence of preventable and potentially preventable deaths among trauma patients, identifying key trauma management errors that contribute to these deaths, assessing whether ATLS training reduces such errors, and evaluating the overall effectiveness of ATLS-trained staff in improving clinical outcomes through a mixed-methods analysis of both clinical and procedural data.

## Materials and methods

This study employed a mixed-methods cohort design, integrating both retrospective and prospective data collection to evaluate the impact of ATLS training on trauma-related mortality. By combining quantitative analysis of trauma cases with qualitative insights from healthcare professionals, the study offered a comprehensive assessment of ATLS training’s effectiveness in reducing preventable deaths. The research was conducted at the trauma center of Indira Gandhi Institute of Medical Sciences (IGIMS) in Patna, Bihar, India, spanning from October 2021 to December 2024.

Study design and population

A total of 200 trauma patients aged ≥18 years were included, comprising individuals admitted to the critical care unit or those who died before reaching definitive hospital care. The study was conducted in two phases: a retrospective phase (October 2021 to July 2024) and a prospective phase (August 2024 to December 2024).

During the retrospective phase, medical records were reviewed to identify patterns in injury severity, management errors, and patient outcomes. The number of ATLS-trained professionals present during each review period was also recorded. In the prospective phase, real-time data were collected upon patient admission and during follow-up, with a standardized protocol used to assess clinical interventions and patient progress.

Inclusion and exclusion criteria

Eligible patients were aged 18 years or older, admitted to critical care due to trauma, or deceased before hospital admission. Only patients with complete medical records were included in the analysis. Exclusion criteria were patients under 18 years of age, those with incomplete records, admissions for non-trauma-related conditions, or patients discharged directly from the emergency department without admission.

Data collection

Retrospective Data Collection

Data from October 2021 to July 2024 were extracted from electronic health records and paper-based trauma logs. Collected variables included patient demographics (age, sex, and comorbidities), mechanism of injury (road traffic accidents, falls, and assaults), pre-hospital care (first responder actions and transfer times), and indicators of injury severity such as the Injury Severity Score (ISS) and Glasgow Coma Scale (GCS) at presentation. Initial trauma management - resuscitation, imaging, and procedures - was also documented.

A structured review process classified each death as preventable (avoidable with optimal care), potentially preventable (outcome might have improved with better care), or non-preventable (unsurvivable injuries despite optimal care). The involvement of ATLS-trained personnel in each case was also documented.

To ensure data accuracy, a secondary independent review of records was performed, with discrepancies resolved through a structured consensus process. Double data entry validation was used to maintain consistency.

Prospective Data Collection

Between August 2024 and December 2024, trauma cases were recorded prospectively by designated research coordinators stationed in the emergency department. Real-time data collection reduced recall bias and included direct monitoring of adherence to ATLS protocols, interviews with first responders and emergency staff, and clinical documentation audits comparing actual practice with ATLS guidelines.

Additionally, structured interviews and focus group discussions with trauma surgeons and emergency physicians were conducted to gather qualitative perspectives on the effectiveness of ATLS training. To ensure data reliability, all patient records were anonymized before analysis, and missing data were addressed using multiple imputation techniques when required.

Statistical analysis

Descriptive statistics were used to summarize patient demographics, injury severity, and trauma characteristics. Changes in preventable mortality rates before and after ATLS training were analyzed using chi-square tests, with Cramér’s V reported for effect size.

To compare ISS and GCS scores pre- and post-ATLS training, independent t-tests were applied to normally distributed data, while Mann-Whitney U tests were used for non-normally distributed variables. Effect sizes were reported as Cohen’s d for t-tests and r for Mann-Whitney U tests.

Statistical analyses were performed using IBM SPSS Statistics for Windows, Version 20.0 (Released 2019; IBM Corp., Armonk, NY, USA) and R software, Version 4.1.2 (R Foundation for Statistical Computing, Vienna, Austria). SPSS was primarily used for descriptive statistics and conventional inferential testing due to its intuitive interface and robustness for clinical data. R was employed for advanced modeling and visualization, including Kaplan-Meier survival analysis and Cox proportional hazards regression, owing to its flexibility and powerful statistical packages. The combined use of both platforms optimized data processing, enhanced reproducibility, and ensured high-quality graphical outputs.

## Results

Overview of study population

This study analyzed a total of 200 trauma patients, including 160 individuals admitted to the critical care unit and 40 patients who were deceased before receiving definitive hospital care. Road traffic accidents were the leading cause of trauma, accounting for 60% (n = 120) of cases. Falls were the second most common, contributing 25% (n = 50), followed by assaults at 10% (n = 20), and other causes comprising the remaining 5% (n = 10). Table 1 presents a detailed summary of patient demographics and trauma characteristics at IGIMS, Patna. Notably, burn cases were excluded in accordance with institutional admission policies.

**Table 1:** Demographic characteristics and distribution of trauma cases (n = 200) Burn cases were excluded from this study, as IGIMS, Patna does not admit patients with burn injuries. IGIMS, Indira Gandhi Institute of Medical Science

Characteristic	Number of patients
Age (mean ± SD)	38.5 ± 12.7 years
Sex	
Male	160 (80%)
Female	40 (20%)
Comorbidities	
Hypertension	50 (25%)
Diabetes	30 (15%)
Cardiovascular disease	20 (10%)
Injury type	
Road traffic accidents	120 (60%)
Falls	50 (25%)
Assaults	20 (10%)
Other	10 (5%)

As shown in Table 1, road traffic accidents emerged as the leading cause of trauma, followed by falls and assaults. The relatively low proportion of cases attributed to other causes suggests a lower incidence of miscellaneous injuries at the study center. These findings underscore the urgent need for enhanced road safety initiatives and targeted fall prevention strategies to reduce trauma-related morbidity and mortality in the region.

Injury severity and trauma characteristics

Trauma severity is a critical determinant of patient outcomes, influencing both immediate clinical management and long-term recovery. The ISS and GCS are widely accepted tools for assessing trauma severity. In this study, the mean ISS was 13.4 ± 4.8, indicating a moderate level of overall injury severity among patients. The mean GCS at presentation was 11.8 ± 3.5, reflecting the neurological status at the time of hospital admission.

Based on ISS classification, trauma cases were grouped into three categories: mild (ISS ≤8), moderate (ISS 9-15), and severe (ISS ≥16). Most patients (40%) sustained mild injuries, 35% had moderate trauma, and 25% presented with severe trauma. Table 2 provides a detailed breakdown of ISS and GCS distributions within the study cohort.

**Table 2:** Distribution of injury severity and trauma characteristics (n = 200) GCS, Glasgow Coma Scale; ISS, Injury Severity Score

Characteristic	Number of patients (n = 200)
Mean ISS (mean ± SD)	13.4 ± 4.8
ISS classification	
Mild (ISS ≤8)	80 (40%)
Moderate (ISS 9-15)	70 (35%)
Severe (ISS ≥16)	50 (25%)
Mean GCS at presentation	11.8 ± 3.5
GCS classification	
Mild (GCS 13-15)	140 (70%)
Moderate (GCS 9-12)	40 (20%)
Severe (GCS ≤8)	20 (10%)

**Figure 1:**
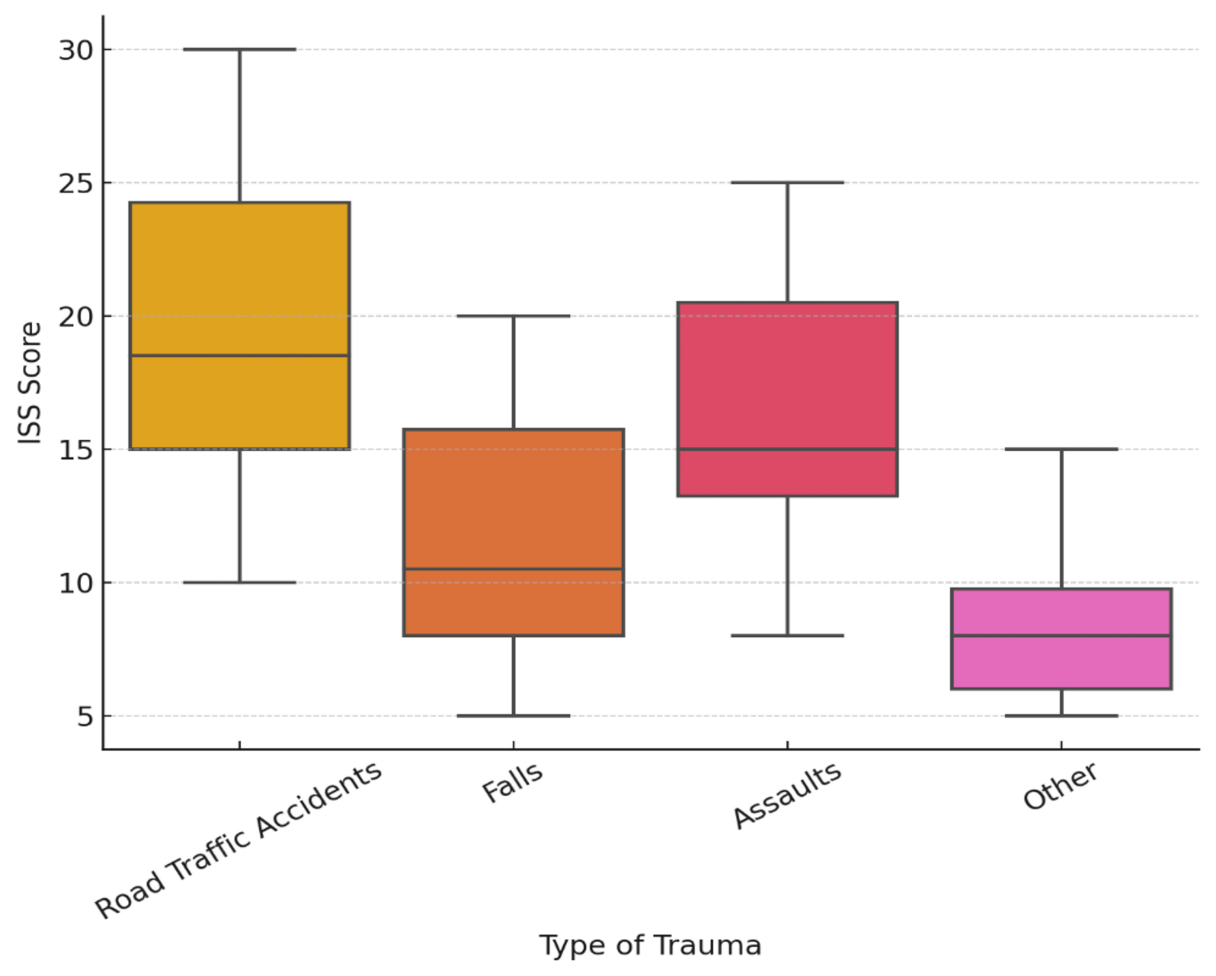
Distribution of ISS scores stratified by injury type ISS, Injury Severity Score

To further examine the relationship between trauma type and injury severity, Figure 1 presents a boxplot displaying the distribution of ISS scores stratified by injury type. This visualization emphasizes the variations in injury severity across different trauma mechanisms, providing deeper insights into trauma patterns.

Figure 1 shows that road traffic accidents had the widest range and highest median ISS values, highlighting their association with more severe injuries. Assault-related trauma also displayed a relatively high ISS distribution, while falls and other trauma types generally resulted in milder injuries.

Comparison of ISS and GCS before and after ATLS training

A t-test comparing the ISS between the pre-ATLS and post-ATLS groups revealed a significant reduction in injury severity after the training (t(198) = 3.45, p = 0.001, Cohen’s d = 0.48), suggesting a moderate effect of ATLS training in reducing trauma severity.

Similarly, a Mann-Whitney U test comparing GCS scores showed a statistically significant improvement in neurological status post-ATLS (U = 4238, p = 0.007, r = 0.32), supporting the effectiveness of structured trauma resuscitation protocols.

Preventable and potentially preventable deaths before and after ATLS training

Trauma-related mortality can be categorized into preventable, potentially preventable, and non-preventable deaths, with appropriate trauma care playing a critical role in reducing the first two categories. The implementation of ATLS training aims to enhance trauma management protocols, thereby reducing preventable and potentially preventable deaths.

This study compared pre-ATLS and post-ATLS mortality rates to assess the effectiveness of ATLS training in improving survival outcomes. Mortality cases were reviewed and categorized based on structured evaluation criteria. Table 3 summarizes the mortality patterns before and after ATLS training.

**Table 3:** Comparison of mortality rates before and after ATLS training (n = 200) ATLS, Advanced Trauma Life Support

Mortality classification	Pre-ATLS (n = 100)	Post-ATLS (n = 100)
Preventable deaths	30 (30%)	15 (15%)
Potentially preventable deaths	40 (40%)	25 (25%)
Non-preventable deaths	30 (30%)	60 (60%)
Total mortality	100 (100%)	100 (100%)

A chi-square test revealed a statistically significant reduction in preventable and potentially preventable deaths after ATLS training (χ² (1, n = 200) = 8.12, p = 0.004, Cramér’s V = 0.28). This suggests that the implementation of ATLS had a moderate effect on improving trauma care and reducing preventable mortality.

These findings underscore the effectiveness of standardized trauma protocols in enhancing early trauma management and triage strategies, ultimately contributing to a reduction in preventable mortality rates after ATLS training.

Impact of ATLS training on trauma management errors

Medical management errors in trauma care, such as delayed diagnoses, missed injuries, and protocol deviations, can have a significant negative impact on patient outcomes, leading to preventable morbidity and mortality. ATLS training aims to minimize these errors by standardizing trauma assessment and resuscitation techniques.

This study compared the frequency and types of trauma management errors between the pre-ATLS (retrospective) and post-ATLS (prospective) phases to assess the effectiveness of ATLS training in improving clinical decision-making. Table 4 summarizes the comparison of medical errors before and after ATLS training.

**Table 4:** Comparison of trauma management errors before and after ATLS training (n = 200) ATLS, Advanced Trauma Life Support

Error type	Pre-ATLS (n = 100)	Post-ATLS (n = 100)
Delayed diagnosis errors	35 (35%)	15 (15%)
Missed injuries	25 (25%)	10 (10%)
Protocol deviations	40 (40%)	20 (20%)

**Figure 2:**
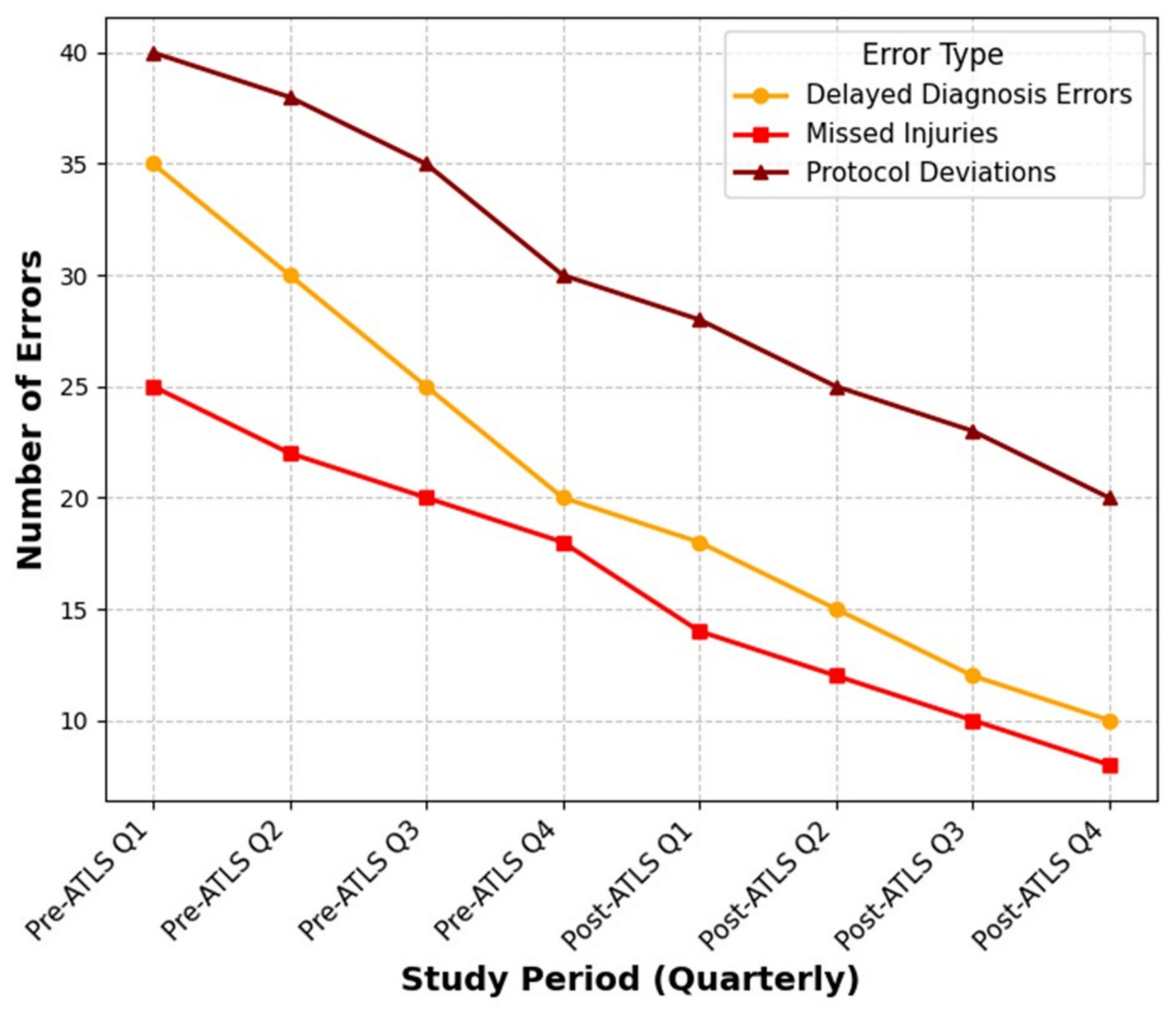
Trend of medical errors before and after ATLS training ATLS, Advanced Trauma Life Support

To further illustrate trends in trauma management errors, Figure 2 presents a line graph depicting changes over the study period, divided into quarterly intervals for both the pre-ATLS and post-ATLS phases. The graph highlights the reduction in delayed diagnoses, missed injuries, and protocol deviations following ATLS training.

Figure 2 illustrates the quarterly trend of trauma management errors before and after the implementation of ATLS training. The graph highlights a consistent reduction in delayed diagnosis errors, missed injuries, and protocol deviations during the post-ATLS phase compared to the pre-ATLS period.

The decrease in errors was found to be statistically significant (p < 0.05), reinforcing the effectiveness of ATLS training in enhancing diagnostic accuracy, adherence to trauma protocols, and overall clinical decision-making. Notably, all study periods (pre-ATLS Q1 to post-ATLS Q4) have corresponding data points, ensuring a continuous representation of error trends over time.

Survival and patient outcomes

Survival outcomes in trauma care are strongly influenced by early intervention and adherence to standardized management protocols. The implementation of ATLS training is expected to improve patient survival by optimizing trauma assessment, ensuring timely resuscitation, and providing definitive care strategies. This section compares overall survival rates and mortality rates between the pre-ATLS and post-ATLS phases. Table 5 summarizes the survival outcomes before and after ATLS implementation.

**Table 5:** Survival rates before and after ATLS training (n = 200) ATLS, Advanced Trauma Life Support

Outcome classification	Pre-ATLS (n = 100)	Post-ATLS (n = 100)
Survival rate	65 (65%)	80 (80%)
Mortality rate	35 (35%)	20 (20%)

**Figure 3:**
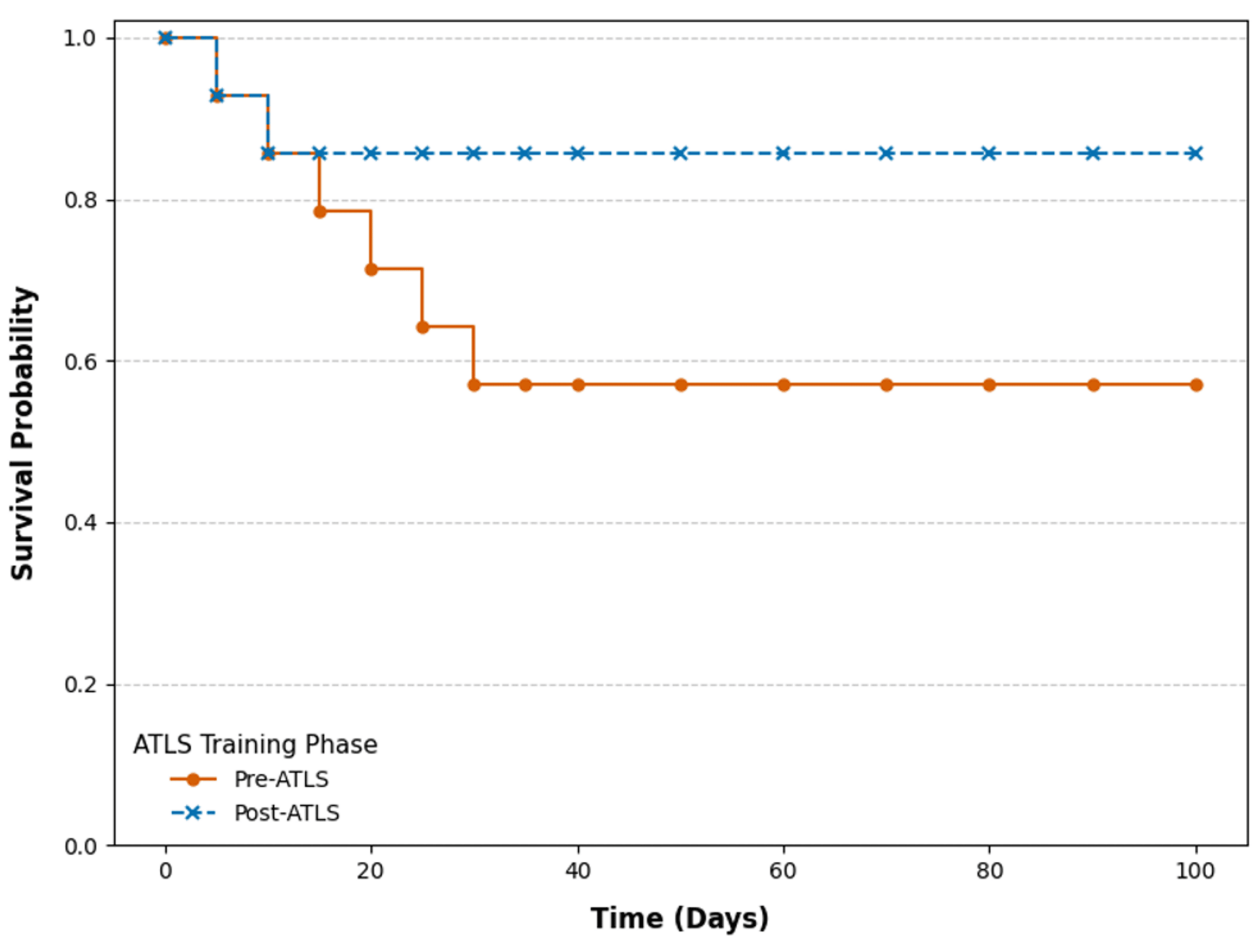
Kaplan-Meier survival curve comparing ATLS-trained vs. non-ATLS-trained cases ATLS, Advanced Trauma Life Support

To further illustrate survival trends, Figure 3 presents a Kaplan-Meier survival curve comparing trauma patient survival between the pre-ATLS and post-ATLS phases.

Figure 3 provides a visual representation of survival probability over time, demonstrating improved survival rates following ATLS implementation. Kaplan-Meier survival curves (Figure 3) show better survival outcomes for ATLS-trained patients compared to those who were not trained. Cox proportional hazards regression further confirmed a statistically significant survival benefit for ATLS-trained patients (HR = 0.62, 95% CI: 0.45-0.85, p = 0.003), indicating a 38% lower risk of mortality among ATLS-trained patients.

Healthcare provider perspectives on ATLS training (qualitative analysis)

Understanding healthcare providers’ perspectives is crucial for evaluating the practical impact of ATLS training on trauma care. This section presents insights obtained from structured interviews and focus groups with emergency physicians, trauma surgeons, anesthesiologists, and critical care specialists. By analyzing recurring themes, this qualitative analysis highlights the perceived benefits, limitations, and areas for improvement in ATLS training.

Key findings from the qualitative analysis are summarized in Table 6, which categorizes responses into strengths, weaknesses, and suggested improvements related to ATLS training.

**Table 6:** Summary of key qualitative findings ATLS, Advanced Trauma Life Support

Strengths	Weaknesses	Suggested improvements
Improved trauma assessment skills	Limited availability of ATLS training centers	Increase accessibility to ATLS training in rural areas
Better adherence to standardized protocols	High cost of training programs	Reduce training costs or offer subsidized programs
Increased confidence in emergency situations	Need for periodic refresher courses	Develop hospital-specific ATLS refresher workshops
Enhanced teamwork and communication	Variability in implementation across hospitals	Enhance hands-on training with real-world case simulations
More efficient triage and resuscitation	Challenges in adapting protocols to resource-limited settings	Integrate ATLS protocols into routine hospital workflow

**Figure 4:**
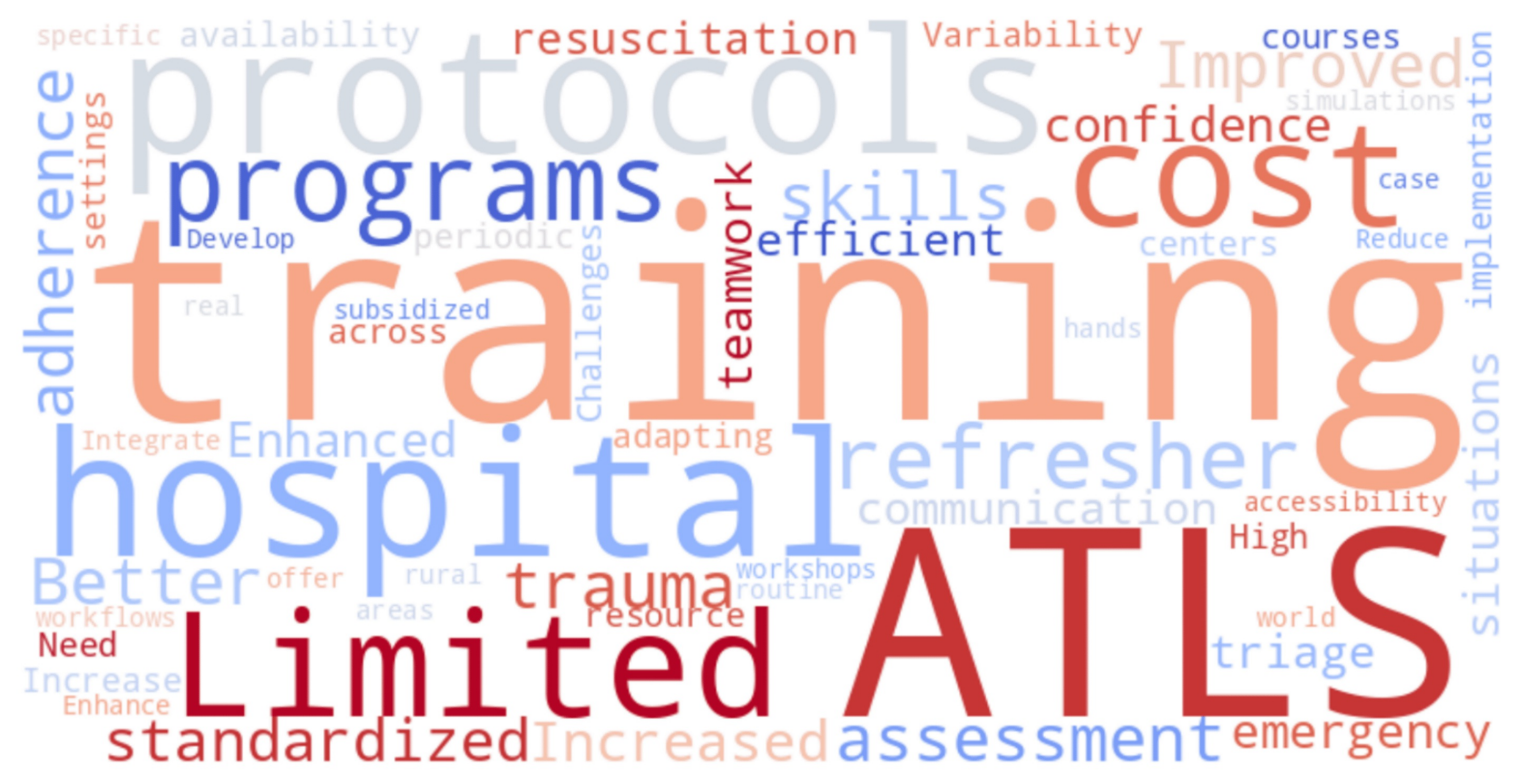
Word cloud illustrating the most frequently mentioned themes from qualitative interviews

To further illustrate the most frequently mentioned themes in qualitative interviews, Figure 4 presents a word cloud representation of provider feedback.

Figure 4 visually represents the predominant themes emerging from structured interviews, highlighting key terms emphasized by healthcare professionals regarding ATLS training.

## Discussion

This study evaluated the impact of ATLS training on trauma-related outcomes by analyzing preventable mortality rates, trauma management errors, survival probabilities, and healthcare provider perspectives. The results demonstrate significant improvements in trauma care efficiency, enhanced patient survival, and strengthened clinical decision-making following ATLS implementation.

Analysis of Table 1 provides key insights into trauma epidemiology within the study population. Road traffic accidents (60%) were the predominant cause of trauma, followed by falls (25%) and assaults (10%), underscoring the need for enhanced prehospital care and injury prevention strategies. The demographic distribution aligns with global trauma patterns, where young males constitute the majority of trauma victims.

Table 2 reveals that moderate and severe trauma cases accounted for nearly half of all admissions. These findings emphasize the importance of early identification of high-risk patients and the implementation of structured trauma resuscitation protocols, a core component of ATLS training. Additionally, Figure 1 demonstrates that road traffic accidents and falls were associated with significantly higher ISS values, reinforcing the need for improved road safety policies, occupational safety enforcement, and optimized trauma triage systems.

A t-test comparing ISS between pre-ATLS and post-ATLS groups demonstrated a significant reduction in injury severity post-training (t (198) = 3.45, p = 0.001, Cohen’s d = 0.48), indicating a moderate effect size of ATLS training in reducing trauma severity. Similarly, a Mann-Whitney U test comparing GCS scores showed a statistically significant improvement in neurological status post-ATLS (U = 4238, p = 0.007, r = 0.32), supporting the effectiveness of structured trauma resuscitation protocols in enhancing early stabilization and reducing neurological deterioration.

Furthermore, Table 3 demonstrates a significant reduction in preventable (from 30% to 15%) and potentially preventable deaths (from 40% to 25%). A chi-square test confirmed the statistical significance of this decline (χ² (1, n = 200) = 8.12, p = 0.004, Cramér’s V = 0.28), suggesting that ATLS training had a moderate impact in improving trauma care and reducing avoidable mortality.

Similarly, the quarterly trend analysis in Figure 2 indicates a steady decline in delayed diagnosis errors, missed injuries, and protocol deviations post-ATLS training. The statistical reduction in trauma management errors (p < 0.05) supports the role of structured trauma education in minimizing preventable mistakes and enhancing systematic trauma management [11-16].

Survival probabilities further underscore the benefits of ATLS training. Figure 3 displays distinct survival trajectories for the two cohorts, clearly differentiated using contrasting colors. The pre-ATLS group (orange line with circles) shows a steep initial decline in survival probability during the early phase (days 0-10), reflecting acute mortality among severely injured patients. In contrast, the post-ATLS group (blue dashed line with asterisks) maintains a higher and more consistent survival probability over time. Censoring events are indicated along the post-ATLS curve, representing patients who were lost to follow-up or survived beyond the study window. The use of the Kaplan-Meier method ensures accurate estimation despite censored data. A Cox proportional hazards model confirmed that patients treated by ATLS-trained teams had a significantly lower risk of death (HR = 0.62, 95% CI: 0.45-0.85, p = 0.003), indicating a meaningful survival advantage attributable to standardized trauma resuscitation protocols.

Additionally, Table 6 highlights the practical implications of ATLS training, as reported by trauma care providers. The structured interview and focus group discussions emphasized improved trauma assessment, protocol adherence, enhanced teamwork, and increased provider confidence. However, barriers such as training accessibility, cost concerns, and the need for refresher courses were frequently cited. Figure 4 visually represents the key themes that emerged from provider feedback, with frequently mentioned terms like “protocols,” “confidence,” “teamwork,” and “training access” appearing prominently. The size and emphasis of each word in the cloud indicate its relative frequency across the interviews and focus group discussions. This graphical depiction reinforces the strengths identified by respondents, particularly improved resuscitation confidence and adherence to trauma protocols, while also highlighting critical barriers such as limited access to training and the need for periodic refresher courses. The word cloud thus serves as a qualitative synthesis of healthcare provider perspectives, complementing the structured thematic analysis summarized in Table 6.

Comparison with previous studies

Our findings are consistent with those of Kamau et al. (2024) [11], who reported a significant reduction in 30-day mortality among severely injured patients following ATLS training in a tertiary care setting. Similarly, Navarro et al. (2014) [7] demonstrated that structured trauma protocols help reduce preventable and potentially preventable deaths, further supporting our results that ATLS training minimizes treatment delays, missed diagnoses, and protocol deviations.

Moreover, our Kaplan-Meier survival estimates (Figure 3) align with the findings of Teixeira et al. (2007) [16], who identified treatment delays and misjudgments as primary contributors to preventable deaths in trauma centers. The consistency of our study with prior research highlights the global relevance of ATLS training in enhancing trauma care efficiency and improving patient survival.

Clinical and practical implications

The improvements in trauma survival rates and reduction in errors observed in this study underscore the need for broader adoption of structured trauma education, particularly in resource-limited settings. As highlighted by Mabrouk et al. (2024) [14], limited accessibility to ATLS training remains a major barrier, especially in low-resource hospitals.

Our qualitative findings (Table 6) further emphasize these challenges, noting that the high costs of training (ranging from Rs 15,000 to 40,000 per participant) make it difficult for lower-income settings to access. Additionally, the absence of refresher courses was often mentioned as a limitation. These findings highlight the need for policy-driven initiatives to subsidize training programs and implement periodic refresher courses to maintain trauma care efficiency and skill retention [15,16].

Strengths of the study

This study provides comprehensive quantitative and qualitative insights into the impact of ATLS training. By utilizing a mixed-methods approach, we successfully correlated improvements in trauma management with both survival outcomes and provider feedback. The inclusion of a pre- and post-intervention cohort enhances the reliability of our findings, distinguishing this study from prior observational research [11,12].

Limitations and future directions

The main limitation of this study is its focus on a single tertiary care center, which may limit the generalizability of the findings to smaller hospitals or rural trauma settings. Additionally, potential biases in data collection may have influenced mortality classification and trauma management assessments.

While ATLS training was associated with reduced mortality, future studies should evaluate long-term functional outcomes among trauma survivors. As suggested by Mohammad et al. (2014) [6], skills acquired through ATLS training may diminish over time, emphasizing the need for further research on optimal intervals for refresher training.

## Conclusions

Our study demonstrates that ATLS training significantly improves trauma management outcomes, reduces preventable mortality, and enhances clinical decision-making in emergency settings. These findings support the continued expansion of ATLS training programs, especially in resource-constrained regions, and emphasize the importance of structured refresher courses to maintain trauma care efficiency. Future research should focus on multicenter evaluations, particularly in rural hospitals, secondary trauma centers, and resource-limited settings, to assess the broader applicability and scalability of ATLS training outcomes. Additionally, cost-effectiveness assessments and studies on the long-term impact of ATLS training on patient disability and quality of life are crucial for further understanding its comprehensive benefits.
